# Preventive measures against HIV among Uganda’s youth: Strategies, implementation, and effectiveness

**DOI:** 10.1097/MD.0000000000040317

**Published:** 2024-11-01

**Authors:** Emmanuel Ifeanyi Obeagu, Getrude Uzoma Obeagu

**Affiliations:** a Department of Medical Laboratory Science, Kampala International University, Ishaka, Uganda; b School of Nursing Science, Kampala International University, Ishaka, Uganda.

**Keywords:** effectiveness, HIV, implementation, preventive measures, strategies, Uganda’s youth

## Abstract

Preventing HIV among Uganda’s youth is a critical public health priority due to the high prevalence of HIV/AIDS and the disproportionate burden of new infections among young people. This paper examines the preventive measures implemented to combat HIV/AIDS among Uganda’s youth, focusing on strategies, implementation efforts, and the effectiveness of interventions. Comprehensive sexuality education programs have been implemented in schools and communities to provide young people with accurate information on HIV transmission, prevention methods, and reproductive health. Condom distribution and promotion campaigns target sexually active youth, while HIV testing and counseling services aim to reach youth in various settings, including health facilities and community outreaches. The implementation of HIV prevention strategies involves collaboration among government agencies, NGOs, healthcare providers, educators, and community leaders. Efforts are made to ensure that prevention programs are culturally appropriate, evidence-based, and responsive to the needs of young people. Despite progress in HIV prevention, challenges persist, including knowledge gaps, stigma, gender inequalities, and socio-economic factors. Continuous monitoring and evaluation are essential to assess the impact of interventions and identify areas for improvement. Recommendations include increasing funding for HIV prevention programs, strengthening policy frameworks, enhancing access to youth-friendly health services, integrating comprehensive sexuality education into school curricula, and fostering community engagement. By addressing these recommendations, Uganda can strengthen its HIV prevention efforts and reduce the incidence of HIV/AIDS among its youth population, ultimately contributing to improved health outcomes and well-being.

## 1. Introduction

HIV/AIDS remains a significant public health challenge in Uganda, with a prevalence rate of 5.7% among adults aged 15 to 49 years. Among the most vulnerable populations are Uganda’s youth, aged 15 to 24 years, who face a heightened risk of HIV infection due to factors such as limited access to sexual health education, economic disparities, and cultural norms surrounding sexuality. The prevention of HIV among Uganda’s youth is imperative not only for individual health but also for broader efforts to combat the HIV epidemic and achieve sustainable development goals. In response to the HIV/AIDS epidemic, Uganda has implemented various preventive measures targeting its youth population. These measures encompass a range of strategies, including comprehensive sexuality education, condom distribution and promotion, HIV testing and counseling services, youth-friendly health facilities, and behavior change communication campaigns. These interventions aim to empower young people with knowledge and skills to make informed decisions about their sexual health, reduce risky behaviors, and access necessary services for HIV prevention and care. The implementation of HIV prevention strategies among Uganda’s youth requires concerted efforts from multiple stakeholders, including government agencies, non-governmental organizations (NGOs), healthcare providers, educators, parents, and community leaders. Collaboration and coordination among these stakeholders are essential to ensure the effective delivery and uptake of preventive interventions, particularly in reaching marginalized and vulnerable youth populations. Despite progress in implementing HIV prevention programs, challenges persist, including funding constraints, limited access to healthcare services in rural areas, and persistent stigma and discrimination associated with HIV/AIDS.^[1–6]^

## 2. Aim

The aim of this report is to comprehensively examine the preventive measures implemented to combat HIV/AIDS among Uganda’s youth, focusing on the strategies employed, the implementation efforts undertaken, and the effectiveness of interventions.

## 3. Rationale

The rationale for conducting this report stems from the urgent need to address the high burden of HIV/AIDS among Uganda’s youth population. Uganda continues to face a significant HIV epidemic, with young people aged 15 to 24 years being particularly vulnerable to infection. The impact of HIV/AIDS on Uganda’s youth is profound, affecting their health, well-being, education, and future prospects. Therefore, there is a pressing need to comprehensively examine the preventive measures implemented to combat HIV/AIDS among Uganda’s youth and assess their effectiveness in reducing HIV incidence and improving health outcomes. Understanding the rationale for preventive measures against HIV/AIDS among Uganda’s youth is crucial for informing evidence-based policies, programs, and interventions. By analyzing existing literature, policy documents, and programmatic data, this report seeks to identify gaps, strengths, and areas for improvement in current HIV prevention efforts targeting Uganda’s youth population. Additionally, the report aims to provide insights into the socio-economic, cultural, and structural factors driving HIV vulnerability among young people and advocate for a holistic and multi-sectoral approach to HIV prevention. Furthermore, the rationale for conducting this report lies in the importance of promoting the health and well-being of Uganda’s youth as a fundamental human right and a key driver of sustainable development. Investing in HIV prevention among youth is not only a public health imperative but also a moral and ethical obligation to safeguard the rights and dignity of young people and ensure their full participation in society. Therefore, by examining the rationale for preventive measures against HIV/AIDS among Uganda’s youth, this report seeks to contribute to the development of evidence-based strategies and policies that effectively address the HIV/AIDS epidemic, promote youth empowerment, and advance the broader goals of health equity and social justice.

## 4. Review methodology

### 4.1. Search strategy

A systematic search strategy was employed to identify relevant literature from electronic databases, including PubMed/MEDLINE, Embase, Scopus, and the World Health Organization Global Health Library. The search strategy utilized a combination of keywords and Medical Subject Headings (MeSH) terms related to HIV/AIDS, youth, prevention strategies, implementation, and effectiveness. Boolean operators (AND, OR) were utilized to combine search terms effectively, and searches were limited to English-language publications.

### 4.2. Inclusion and exclusion criteria

Studies, policy documents, and programmatic reports were included in the review if they met the following criteria:

1. Pertained to HIV prevention efforts targeting youth (aged 15 to 24 years) in Uganda.
2. Reported on preventive measures, strategies, or interventions aimed at reducing HIV incidence among Uganda’s youth population.
3. Included data on implementation efforts, effectiveness, outcomes, or impact of HIV prevention interventions.
4. Published in peer-reviewed journals, government reports, international agency reports, or reputable organizational websites. Studies were excluded if they:
5. Did not focus on HIV prevention among youth in Uganda.
6. Were duplicates, abstracts, conference proceedings, editorials, or commentaries without original data.
7. Were not available in full-text format or were not written in English.

### 4.3. Study selection

Two independent reviewers screened titles and abstracts of identified studies to determine their eligibility for inclusion. Full-text articles, policy documents, and reports were retrieved for further assessment if they met the inclusion criteria or if eligibility could not be determined based on title and abstract alone. Discrepancies between reviewers were resolved through consensus or consultation with a third reviewer.

### 4.4. Data extraction and synthesis

Data extraction was conducted using a standardized data extraction form to capture relevant information from included studies, policy documents, and reports. Extracted data included study characteristics, study design, sample population, intervention details, implementation strategies, outcomes, and findings. Data synthesis involved summarizing and analyzing extracted information to identify common themes, trends, and gaps in the literature related to HIV prevention among Uganda’s youth population.

### 4.5. Quality assessment

The quality of included studies, policy documents, and reports was assessed using appropriate tools based on study design and methodology. Studies were evaluated for methodological rigor, risk of bias, and relevance to the review objectives. Quality assessment was conducted independently by 2 reviewers, and discrepancies were resolved through discussion and consensus.

### 4.6. Burden of HIV among youths of the entire world

The burden of HIV among youths globally remains a significant public health challenge, with varying prevalence rates and complexities in prevention and treatment efforts across different regions.^[7]^ Youth, defined by the World Health Organization as individuals between the ages of 15 and 24, face a substantial burden of HIV/AIDS in many parts of the world.^[8]^ Epidemiological data reveals a diverse landscape of HIV prevalence among youths, with rates influenced by social, economic, cultural, and behavioral factors.^[9]^ In some regions, such as sub-Saharan Africa and parts of Southeast Asia, the burden of HIV among youths is particularly high.^[10]^ Factors contributing to this burden include limited access to comprehensive sexual education, stigma, discrimination, poverty, gender inequalities, and barriers to healthcare services. Behavioral patterns, including early sexual debut, inconsistent condom use, and engaging in risky sexual behaviors, also contribute significantly to the burden of HIV among young people worldwide.^[11]^

While progress has been made in certain areas with increased access to antiretroviral treatment and improved prevention strategies, many regions continue to experience challenges in reducing HIV transmission among youths.^[12]^ In addition to the direct impact on physical health, the burden of HIV affects youths’ educational opportunities, employment prospects, and overall well-being, impacting their future and societal development.^[13]^ Efforts to address the burden of HIV among youths globally require a comprehensive approach that integrates education, prevention, testing, treatment, and support services.^[14]^ Strategies should encompass culturally sensitive interventions, access to youth-friendly healthcare, empowerment programs, and community engagement to effectively reach and support young populations. Promoting awareness, reducing stigma, fostering supportive environments, and empowering young individuals with accurate information and resources are crucial in mitigating the burden of HIV among youths worldwide.^[15]^ Additionally, enhancing access to affordable and quality healthcare services remains a fundamental aspect of addressing the challenges faced by young people affected by HIV/AIDS on a global scale.

### 4.7. Burden of HIV among African youths

The burden of HIV among African youths remains a significant public health concern across the continent, with high prevalence rates and substantial challenges in prevention and treatment efforts.^[16]^ Epidemiological data underscores the disproportionate impact of HIV/AIDS on African youths aged 15 to 24 years.^[17]^ Despite progress in some regions, many countries continue to face a considerable burden of new HIV infections among young people.^[18]^ Factors contributing to this burden vary widely across different countries and regions but often include limited access to comprehensive sexual education, cultural norms, gender disparities, poverty, and barriers to healthcare services. In many African countries, HIV prevalence among young people is notably higher compared to other age groups.^[19]^ Vulnerability to HIV transmission is influenced by various socio-economic factors, including unemployment, lack of access to healthcare, migration, and social inequality.^[20]^ Additionally, behavioral patterns such as early sexual debut, inconsistent condom use, and multiple sexual partnerships contribute significantly to the high rates of HIV infections among African youths.^[21]^ The burden of HIV among African youths not only affects their physical health but also has broader socio-economic implications.^[22]^ It impacts educational attainment, employment opportunities, and overall well-being, hampering the potential and productivity of a vital demographic segment crucial for the continent’s development. Efforts to address the burden of HIV among African youths require multifaceted strategies that encompass comprehensive sexual education, access to HIV testing and treatment, youth-friendly healthcare services, empowerment programs, and targeted interventions tailored to the diverse cultural and social contexts within African countries.^[23]^ Additionally, combating stigma and discrimination associated with HIV/AIDS, promoting gender equality, and empowering young people with knowledge, resources, and supportive environments are crucial in reducing the burden of HIV among African youths.^[24]^ Understanding the multifaceted nature of this burden, along with the socio-economic and cultural factors contributing to it, is fundamental in formulating and implementing effective prevention, treatment, and support initiatives that can mitigate the impact of HIV/AIDS on African youths and foster healthier and more empowered communities.

### 4.8. Epidemiology of HIV among the youths of Uganda

HIV/AIDS remains a significant public health challenge in Uganda, particularly among young people aged 15 to 24 years. This demographic is highly vulnerable due to various social, economic, and behavioral factors that heighten their risk of HIV infection. Despite Uganda’s commendable efforts in curbing the HIV epidemic, the youth continue to bear a disproportionate burden of new infections. This section delves into the epidemiology of HIV among Uganda’s youth, examining the prevalence, incidence rates, associated risk factors, and gender disparities that define the landscape of HIV transmission in this group.^[25,26]^ Uganda has made significant progress in reducing overall HIV prevalence, but youth remain particularly affected. According to the Uganda Population-Based HIV Impact Assessment (UPHIA) report and data from the Uganda AIDS Commission, the prevalence of HIV among young people is estimated to be around 2.1% as of 2020. However, the prevalence rate varies considerably by gender, with young women bearing a higher burden of infection compared to their male counterparts.^[27,28]^ The gender disparity is stark, with young women aged 15 to 24 being more than twice as likely to contract HIV as young men. This is largely attributed to factors such as early sexual debut, gender-based violence, and transactional sex, which disproportionately affect females. Young men, on the other hand, tend to have lower prevalence rates but are still at risk due to risky sexual behaviors such as inconsistent condom use and having multiple sexual partners.^[29]^ HIV incidence – the rate of new infections – among youth remains a concern despite Uganda’s progress in reducing the overall rate of new infections. Uganda’s youth account for a significant proportion of new HIV cases, with estimates suggesting that 40% to 50% of all new infections occur in individuals aged 15 to 24 years. This high rate of new infections highlights the need for targeted interventions aimed at this age group.^[30]^ The incidence rates are particularly high in urban areas and regions with low access to education and healthcare services. Urbanization has been associated with increased risk-taking behaviors, such as higher rates of unprotected sex, drug and alcohol use, and exposure to HIV-positive individuals. In contrast, rural areas, though having lower incidence rates, face challenges related to access to preventive services like condoms, HIV testing, and antiretroviral therapy (ART).^[31]^

Young people in Uganda often engage in sexual activity at an early age, sometimes before they are equipped with the knowledge or resources to protect themselves. Early sexual debut is associated with a higher risk of contracting HIV due to limited access to contraceptives and sexual health education, as well as increased exposure to older sexual partners, who are more likely to be HIV positive.^[31]^ The practice of having multiple sexual partners is common among Ugandan youth, especially males, and is a significant risk factor for HIV transmission. Concurrent sexual relationships increase the likelihood of spreading the virus within networks of partners, making HIV more difficult to control.^[32]^ Poverty and economic hardships lead many young women and men to engage in transactional sex – exchanging sex for money, gifts, or other forms of support. This behavior is particularly risky because it often involves older partners who may have higher HIV prevalence rates, as well as inconsistent condom use. Despite awareness campaigns, the use of condoms among Ugandan youth remains inconsistent. Cultural stigma around condom use, limited access to condoms in rural areas, and misconceptions about condom efficacy contribute to the low levels of use, particularly among young women. Gender-based violence is prevalent in Uganda and is a significant driver of HIV transmission, especially among young women. Coercive sexual relationships, physical violence, and rape expose young women to higher risks of contracting HIV, as these situations often involve unprotected sex and inability to negotiate safer sexual practices. Alcohol and drug use are increasingly common among Uganda’s youth, particularly in urban areas. Substance abuse is closely linked to risky sexual behavior, including unprotected sex and multiple sexual partners. Intoxication impairs judgment and reduces the likelihood of using condoms, thereby increasing the risk of HIV transmission.^[33]^

Gender plays a crucial role in the epidemiology of HIV among Ugandan youth. Young women are disproportionately affected by the epidemic compared to young men, primarily due to social, economic, and cultural inequalities. The prevalence of HIV among young women is significantly higher than among their male counterparts. This is largely driven by early sexual debut, transactional sex, and relationships with older men, who are more likely to be HIV positive. Gender-based violence and lack of autonomy in sexual decision-making further exacerbate young women’s vulnerability. While the prevalence of HIV among young men is lower than among women, they still face significant risks, particularly due to behaviors such as multiple sexual partnerships and low condom use. In some cases, young men’s reluctance to seek healthcare services and testing contributes to higher HIV transmission rates within their communities.^[34]^ HIV prevalence among Uganda’s youth also varies by region. The highest rates are observed in urban areas, particularly in the central region, which includes Kampala. Urbanization, coupled with high population density and increased opportunities for risky behaviors, has led to higher HIV prevalence in cities and towns. In contrast, rural areas tend to have lower prevalence rates but face challenges related to access to healthcare services, education, and HIV prevention tools. Regions with high rates of poverty and limited access to education also see higher rates of HIV transmission among young people. This is particularly evident in the northern and eastern regions of Uganda, where youth often face economic hardships that increase their likelihood of engaging in risky sexual behaviors.^[30,31]^ HIV testing and treatment coverage among Ugandan youth has improved over the years, but gaps remain. According to the Uganda Demographic and Health Survey (UDHS), many young people are still reluctant to get tested for HIV due to stigma, fear of positive results, and lack of youth-friendly services. Young women are more likely to test for HIV than young men, which may partly explain the higher reported prevalence among females. Access to antiretroviral therapy (ART) has expanded in recent years, with the government and international organizations working to provide free ART to those who need it. However, challenges such as adherence to treatment, especially among young people who face social stigma or lack family support, limit the effectiveness of these programs.^[32]^ Uganda has developed youth-friendly health services that aim to provide non-judgmental, accessible, and confidential HIV testing and treatment services to young people. These centers are designed to meet the unique needs of youth, offering sexual health education, counseling, and access to prevention tools like condoms and PrEP. Comprehensive sexual education (CSE) programs are vital in equipping young people with knowledge about HIV transmission and prevention. However, these programs face opposition from conservative cultural and religious groups, leading to inconsistent implementation across the country. The government and NGOs have focused on distributing condoms in schools, universities, and communities to increase access among young people. Awareness campaigns also aim to normalize condom use and dispel myths associated with it. Pre-Exposure Prophylaxis (PrEP) has become an important tool for preventing HIV among high-risk youth populations. While uptake has been slow, efforts are being made to increase awareness and access to PrEP for youth in Uganda.^[33,34]^

### 4.9. Burden of HIV in Uganda youths

The burden of HIV among Uganda’s youth remains a significant and pressing issue, posing substantial challenges to public health and social well-being.^[35]^ The youth demographic, comprising individuals between the ages of 15 and 24, faces a disproportionate burden of HIV infection within the country.^[25]^ Epidemiological data continues to depict a concerning picture, indicating a higher prevalence of HIV among Uganda’s youth compared to other age groups.^[26]^ Despite progressive efforts in HIV prevention and intervention programs, the youth demographic still bears a substantial portion of new infections.^[27]^ Various studies and surveys highlight the prevalence rates, revealing that a considerable percentage of new HIV infections occur within this age cohort.^[28–30]^ Factors contributing to this burden include limited access to comprehensive sexual education, socio-economic disparities, cultural norms, gender inequalities, and barriers to healthcare services tailored to youth-specific needs.^[31]^ Moreover, the burden of HIV among Uganda’s youth is not merely quantitative but also qualitative, impacting various aspects of their lives.^[32]^ Stigma, discrimination, and psychosocial challenges further compound the burden of living with HIV among young individuals, affecting their mental health, educational opportunities, and social integration.^[33]^ This burden significantly impedes the realization of the potential and aspirations of Uganda’s youth population.^[34]^ It not only affects individual health but also hampers national development efforts, as young people represent a vital demographic for economic growth and social progress. Addressing the burden of HIV among Uganda’s youth requires a comprehensive approach that tackles the root causes, promotes access to quality healthcare and education, empowers young individuals with knowledge and resources, and combats societal stigma and discrimination associated with HIV/AIDS.^[14]^ Understanding the magnitude and impact of this burden is pivotal in designing and implementing effective strategies tailored to the specific needs of Uganda’s youth, aiming to alleviate the burden of HIV and enable them to lead healthy, fulfilling lives.

### 4.10. Comprehensive preventive strategies

Comprehensive preventive strategies play a pivotal role in mitigating the spread of HIV among youths, encompassing a multifaceted approach that addresses various factors influencing transmission.^[36]^ These strategies aim not only to prevent new infections but also to empower young individuals with knowledge, resources, and support to make informed decisions regarding their sexual health. Several key preventive strategies include:

### 4.11. Comprehensive sexual education

CSE is an essential component of preventive strategies aimed at reducing HIV transmission among youths.^[37]^ It encompasses a holistic approach to providing young individuals with accurate, age-appropriate, and culturally sensitive information about sexuality, relationships, reproductive health, and HIV/AIDS. Providing scientifically sound information about reproductive anatomy, contraception, sexually transmitted infections (STIs), including HIV/AIDS, and their prevention.^[38]^ Teaching about healthy relationships, consent, communication skills, and respect for oneself and others, fostering the development of positive social skills.^[39]^ Addressing issues of gender equality, gender-based violence, LGBTQ + inclusivity, and cultural diversity, ensuring inclusivity and sensitivity to diverse identities and experiences.^[40]^ Equipping young individuals with decision-making skills, critical thinking, and assertiveness to make informed choices regarding their sexual health and behavior.^[41]^ Educating about reproductive health, rights, and responsibilities, including information on family planning, pregnancy prevention, and access to healthcare services.^[42]^ Integrating life skills education, values clarification, and ethical considerations to foster personal development and responsible behavior.^[43]^ Empowers youths to make informed decisions, leading to reduced risky sexual behaviors, such as early sexual debut, unprotected sex, and multiple partners.^[44]^ Provides accurate information about HIV/AIDS and STIs, increasing awareness and dispelling myths or misconceptions.^[45]^ Promotes positive sexual health by encouraging safer sexual practices, promoting contraception, and facilitating early detection through regular testing.^[46]^ Enhances self-esteem, confidence, and communication skills, enabling youths to assertively negotiate safer sex and navigate relationships.^[47]^ Challenges stigma and discrimination associated with HIV/AIDS, gender, and sexual orientation, fostering a more inclusive and tolerant society.^[48]^ Helps create a healthier and more responsible generation, positively impacting societal attitudes toward sexual health and relationships.^[49]^ Comprehensive sexual education is most effective when integrated into formal education curricula, complemented by community-based programs, and supported by parents, educators, healthcare providers, and policymakers. Its implementation and continuous evaluation play a crucial role in promoting sexual health and reducing HIV transmission among youths.

### 4.12. Accessible HIV testing and counseling

Accessible HIV testing and counseling are fundamental components of HIV prevention and control strategies, especially among youths, offering numerous benefits in reducing transmission rates and improving health outcomes.^[50]^ Ensuring convenient access to HIV testing services in various settings such as healthcare facilities, community centers, schools, and through mobile clinics, promoting easy and non-stigmatizing access for youths.^[51]^ Providing confidential and private testing environments that respect the confidentiality of test results, allowing young individuals to seek testing without fear of stigma or discrimination.^[52]^ Offering comprehensive information and counseling before testing, explaining the testing process, discussing risks, and addressing concerns about HIV transmission and prevention.^[50]^ Providing a range of testing options, including rapid tests, self-testing kits, and traditional laboratory-based tests, catering to the preferences and comfort levels of young individuals.^[53]^ Ensuring post-test counseling for individuals receiving their results, providing emotional support, explaining the implications of test results, and facilitating linkage to care and treatment services for those testing positive.^[54]^ Using testing as an opportunity for education and awareness about HIV/AIDS, encouraging regular testing for sexually active individuals and those engaging in high-risk behaviors.^[55]^

Facilitating early diagnosis allows for early initiation of antiretroviral therapy (ART), leading to improved health outcomes and reducing the risk of HIV transmission to others. Offers an opportunity for risk assessment and behavior change counseling, encouraging safer sexual practices and reducing high-risk behaviors among young individuals. Normalizing HIV testing helps challenge stigma and discrimination associated with HIV/AIDS, promoting a more supportive environment for those seeking testing and treatment.^[56–67]^ Plays a crucial role in prevention efforts by identifying new cases, preventing further transmission, and contributing to the control of the epidemic. Enhances health literacy among young individuals, encouraging proactive engagement in their sexual health and promoting regular testing as a preventive measure. Accessible HIV testing and counseling services, when coupled with effective linkage to care and support services, play a critical role in reducing HIV transmission rates, improving health outcomes, and empowering young individuals to take charge of their sexual health.^[68]^ Ensuring their accessibility, confidentiality, and integration into broader health services are essential in addressing HIV among youths.

### 4.13. Condom distribution and promotion

Condom distribution and promotion are essential components of HIV prevention strategies, especially among youths, aiming to reduce the risk of HIV transmission and other sexually transmitted infections (STIs).^[69]^ Ensuring widespread availability of condoms through various distribution channels, including healthcare facilities, community centers, educational institutions, vending machines, and outreach programs targeting youths.^[70]^ Providing high-quality condoms at an affordable or no-cost basis, ensuring accessibility to economically disadvantaged youths.^[71]^ Conducting targeted campaigns and educational programs to promote condom use, emphasizing their effectiveness in preventing HIV/STIs and addressing misconceptions or stigma associated with condom use.^[72]^ Educating youths on the correct use of condoms, including proper storage, handling, and usage instructions, to ensure effectiveness in preventing transmission.^[73]^ Addressing myths and misconceptions surrounding condom use, such as concerns about reduced pleasure or effectiveness, through accurate information and communication.^[74]^ Implementing innovative and youth-friendly approaches, such as peer education, social media campaigns, and interactive workshops, to engage and encourage condom use among young individuals.^[75]^ Condoms act as a barrier, effectively reducing the risk of HIV transmission and other STIs during sexual intercourse, providing a cost-effective method of prevention.^[69]^ Encourages young individuals to take responsibility for their sexual health, empowering them to make informed decisions and negotiate safer sex practices.^[76]^ Provides dual protection against unintended pregnancies and sexually transmitted infections, offering a comprehensive approach to sexual health.^[77]^ Contributes to reducing HIV transmission rates and supports broader efforts in controlling the spread of the epidemic.^[78]^ Encourages the adoption of safer sexual behaviors, such as consistent and correct condom use, thereby reducing the risk of acquiring or transmitting HIV/STIs.^[79]^ Condom promotion can contribute to discussions around gender equality, emphasizing shared responsibility in sexual health and encouraging open communication between partners.^[80]^ Condom distribution and promotion programs, when coupled with comprehensive sexual education and access to testing and treatment services, play a crucial role in reducing the transmission of HIV and STIs among youths.^[81]^ Continuously promoting their accessibility, correct use, and normalization within sexual health discussions are key to their effectiveness in prevention efforts.

### 4.14. Behavior change communication

Behavior change communication (BCC) is a critical approach in HIV prevention strategies, particularly among youths, focusing on influencing attitudes, beliefs, and behaviors related to sexual health.^[82]^ It involves tailored communication efforts aimed at promoting positive behavior change and reducing risky behaviors associated with HIV transmission. Tailoring messages to specific target audiences, considering cultural, social, and demographic factors to ensure relevance and resonance among youths.^[83]^ Utilizing various communication channels such as mass media, social media, peer education, community outreach, workshops, and interactive sessions to disseminate information.^[84]^ Emphasizing positive behaviors, including condom use, delaying sexual debut, reducing multiple partners, communication skills, and seeking HIV testing and treatment.^[85]^ Addressing misconceptions, stigma, and cultural norms that may contribute to risky behaviors, promoting accurate information and dispelling myths.^[86]^ Engaging young individuals actively in discussions, decision-making, and creative activities to encourage ownership and participation in behavior change initiatives.^[87]^ Fostering life skills, critical thinking, assertiveness, and self-efficacy among youths to enable them to make informed decisions about their sexual health.^[88]^

Enhancing awareness and knowledge about HIV/AIDS, transmission risks, prevention methods, and available services among young individuals.^[89]^ Promoting changes in attitudes and behaviors, encouraging safer sexual practices, and reducing high-risk behaviors associated with HIV transmission.^[90]^ Empowering youths with information and skills to make informed choices about their sexual health, fostering autonomy and responsibility.^[91]^ Challenging stigma and discrimination associated with HIV/AIDS, gender, and sexual orientation through positive messaging and inclusive communication.^[92]^ Influencing societal norms by engaging communities, promoting open dialogue, and encouraging positive norms surrounding sexual health and HIV prevention.^[46]^ Contributing to long-term changes in behaviors and attitudes toward HIV prevention, fostering a healthier and more responsible generation.^[93]^ Behavior Change Communication, when integrated into comprehensive prevention programs, plays a vital role in shaping attitudes and behaviors related to HIV prevention among youths.^[94]^ Customizing messages to resonate with young individuals and engaging them actively in the process are key elements in its effectiveness in promoting positive behavior change.

### 4.15. Youth-friendly health services

Youth-friendly health services (YFHS) are essential in providing accessible, nondiscriminatory, and comprehensive healthcare tailored to the specific needs of young individuals, including sexual and reproductive health (SRH) services.^[95]^ These services aim to create a supportive environment that encourages youths to seek healthcare and information related to their sexual health. Ensuring easy access to healthcare facilities, preferably located in convenient locations, offering extended hours, and providing services free from financial barriers.^[96]^ Respecting the confidentiality and privacy of young individuals, including maintaining confidentiality in counseling and health records.^[97]^ Adopting a non-judgmental and respectful attitude towards youths, irrespective of their sexual orientation, gender identity, or behavior.^[98]^ Offering a range of services related to SRH, including HIV testing, counseling, contraception, STI screening, pregnancy counseling, and information on healthy relationships.^[99]^ Engaging youths in the design and evaluation of services, encouraging their active participation, and considering their perspectives in service provision.^[100]^ Creating a welcoming and comfortable atmosphere within healthcare facilities, with trained staff and youth-friendly materials, ensuring a positive experience for young clients.^[101]^

Encourages youths to seek healthcare services, reducing barriers that prevent them from accessing essential health information and treatment.^[102]^ Facilitates early detection and treatment of health issues, including HIV/STIs, leading to improved health outcomes among young individuals.^[103]^ Fosters a sense of empowerment and trust among youths, encouraging them to engage with healthcare providers and take charge of their health.^[104]^ Provides opportunities for counseling and guidance on safer sexual practices, leading to a reduction in risky behaviors associated with HIV transmission.^[105]^ Encourages regular visits and fosters continuity of care, ensuring ongoing support and follow-up for youths seeking healthcare services.^[106]^ Promotes a positive experience with healthcare services, leading to greater satisfaction among young individuals and encouraging them to return for future care.^[107]^ Youth-friendly health services play a vital role in promoting access to essential healthcare, including SRH services, for young individuals.^[108]^ Ensuring the provision of nondiscriminatory, confidential, and comprehensive services that address the specific needs of youths is crucial in improving their overall health and well-being.

### 4.16. Community engagement and peer education

Community engagement and peer education are integral strategies in HIV prevention among youths, leveraging community-based approaches and peer-to-peer interactions to disseminate accurate information, foster behavioral change, and create supportive environments.^[51]^ Involving trained peers as educators or facilitators to deliver HIV prevention messages, conduct workshops, and engage with fellow youths in community settings.^[109]^ Organizing interactive sessions, workshops, and group discussions led by peers to share experiences, provide information, and encourage dialogue on sexual health topics.^[46]^ Engaging community members, leaders, and stakeholders in advocacy, awareness campaigns, and community-based initiatives aimed at HIV prevention among youths.^[110]^ Adapting educational materials and messages to be culturally relevant, acknowledging diverse beliefs, values, and practices within the community.^[111]^ Establishing support networks and peer support groups where youths can access information, guidance, and support from peers who act as positive role models.^[112]^ Utilizing innovative approaches such as theater, art, music, or digital media campaigns to engage and disseminate HIV prevention messages effectively.^[113]^ Peer educators serve as relatable sources of information, fostering trust and credibility among youths, making the information more accessible and engaging.^[114]^ Utilizing peers allows for broader outreach within communities, enabling messages to reach marginalized or difficult-to-reach populations among youths.^[115]^ Peer-led initiatives create a supportive environment where young individuals can access guidance, support, and information from peers who understand their experiences and challenges.^[116]^ Peer education encourages positive behavior change by promoting safer sexual practices, reducing stigma, and influencing positive norms within the community.^[117]^ Engaging the community fosters ownership, encouraging collective action, and promoting advocacy efforts to address broader social determinants impacting HIV prevention.^[118]^ Building local capacity through peer education programs fosters sustainability, creating a lasting impact within communities beyond short-term interventions.^[119]^ Community engagement and peer education play a crucial role in complementing formal education and health services, providing a supportive and empowering environment for young individuals to access accurate information, discuss concerns openly, and make informed decisions about their sexual health. Integrating these strategies within broader HIV prevention initiatives enhances their effectiveness in reaching and positively influencing youths.^[120]^

### 4.17. Empowerment and life skills programs

Empowerment and life skills programs for youths are pivotal in HIV prevention strategies, equipping young individuals with the knowledge, skills, and resources necessary to make informed decisions, navigate challenges, and protect their sexual health.^[121]^ Offering programs that focus on developing essential life skills such as communication, decision-making, negotiation, critical thinking, and problem-solving.^[122]^ Promoting self-esteem, resilience, and confidence among youths to enable them to make assertive decisions regarding their sexual health and relationships.^[123]^ Providing comprehensive sexual education that empowers youths with accurate information about HIV/AIDS, reproductive health, contraception, and healthy relationships.^[123]^ Teaching coping strategies, stress management techniques, and emotional regulation to help youths deal with peer pressure, stress, and challenges effectively.^[121]^ Encouraging goal setting, future planning, and encouraging positive aspirations among young individuals to motivate them to take charge of their future. Addressing gender inequalities, promoting gender equality, and empowering young individuals to challenge stereotypes and promote respectful relationships.^[121]^

Equips young individuals with the skills to make informed decisions regarding their sexual health, relationships, and behaviors. Enhances communication skills, enabling youths to communicate effectively, assertively, and responsibly in relationships and sexual encounters.^[124]^ Builds resilience and reduces risky behaviors by imparting coping strategies and problem-solving skills to handle peer pressure and challenging situations.^[125]^ Fosters a sense of self-efficacy and agency among youths, empowering them to take control of their health and well-being.^[121]^ Promotes positive behavioral change by encouraging safer sexual practices, reducing risky behaviors, and fostering responsible decision-making.^[126]^ Equips youths with skills that extend beyond sexual health, contributing to their personal development, empowerment, and success in various aspects of life. Empowerment and life skills programs are essential components of comprehensive HIV prevention efforts, as they empower youths with the tools and capabilities to navigate challenges, make informed decisions, and lead healthy and fulfilling lives.^[127]^ Integrating these programs into educational curricula and community initiatives can significantly contribute to reducing HIV transmission among young individuals and promoting their overall well-being.

### 4.18. Addressing structural and socioeconomic factors

Addressing structural and socioeconomic factors is crucial in comprehensive HIV prevention strategies, particularly among youths, as these factors significantly influence vulnerability to HIV transmission.^[2]^ Implementing programs that focus on poverty alleviation, economic empowerment, and creating opportunities for disadvantaged youths to reduce their vulnerability to HIV.^[121]^ Promoting access to quality education and literacy programs, as education correlates with reduced HIV risk and improved health outcomes among youths.^[128]^ Addressing gender inequalities, empowering women and girls, promoting their rights, and challenging harmful gender norms that contribute to HIV vulnerability.^[2]^ Ensuring equitable access to healthcare services, including SRH services, HIV testing, treatment, and care, particularly for marginalized populations.^[129]^ Establishing social protection systems, support networks, and safety nets for vulnerable youths, including orphans, street children, and those affected by conflict or displacement. Combating stigma and discrimination associated with HIV/AIDS, sexual orientation, and gender identity to create an enabling environment for seeking healthcare and support.^[48]^ Advocating for legal reforms and policies that protect the rights of youths, address discrimination, and promote inclusive and supportive environments.

Addresses underlying determinants that make youths more vulnerable to HIV transmission, reducing their risk through improved socio-economic conditions.^[130]^ Enhances access to healthcare, education, and support services, reducing barriers that prevent youths from seeking essential health information and treatment. Fosters empowerment, agency, and resilience among youths, enabling them to make informed decisions about their health and future.^[131]^ Contributes to reducing inequalities, particularly gender-based disparities, empowering marginalized groups, and creating a more inclusive society. Strengthens communities by addressing social determinants, leading to healthier, more resilient, and supportive environments for youths. Provides sustainable solutions that address root causes, contributing to long-term HIV prevention efforts among youths.^[14]^ Addressing structural and socioeconomic factors requires a multi-sectoral approach involving governments, NGOs, community organizations, and policymakers. By targeting these factors, HIV prevention efforts can create a more enabling environment for youths, reducing their vulnerability to HIV transmission and promoting their overall health and well-being.^[132]^

### 4.19. Innovative technology and media campaigns

Innovative technology and media campaigns play a significant role in HIV prevention among youths, leveraging digital platforms, social media, and creative campaigns to disseminate information, raise awareness, and promote behavior change.^[133]^ Utilizing popular social media platforms (e.g., Facebook, Instagram, TikTok) to disseminate HIV prevention messages, educational content, and interactive campaigns targeted at youths.^[134]^ Developing mobile apps that provide access to information, tools for HIV prevention, reminders for testing, and resources for safer sexual practices.^[135]^ Creating online platforms, websites, or portals offering accurate and user-friendly information about HIV/AIDS, sexual health, and available services.^[136]^ Developing engaging content such as videos, animations, quizzes, and infographics that capture youths’ attention and effectively deliver HIV prevention messages.^[137]^ Establishing online peer support groups, forums, or chat platforms where youths can engage, share experiences, and access information anonymously. Organizing webinars, virtual workshops, or live sessions to provide interactive discussions, expert talks, and educational events on HIV prevention.^[138]^

Reaches a broader audience of youths, including those in remote areas, using digital platforms accessible via smartphones and the internet. Captures youths’ attention through interactive and engaging content, encouraging active participation and sharing of information within their social networks. Enables real-time dissemination of updated information and tailored messaging to address specific needs and behaviors among different youth demographics. Challenges stigma and misconceptions surrounding HIV/AIDS through informative and relatable content shared on digital platforms.^[139]^ Influences behavior change by providing accurate information, promoting safer sexual practices, and fostering informed decision-making among youths. Empowers youths by providing access to resources, tools, and support networks for HIV prevention and sexual health.^[140]^ Utilizing innovative technology and media campaigns offers an effective means to engage and educate youths on HIV prevention, leveraging platforms that are integral to their daily lives. By leveraging digital tools and creative content, these campaigns can effectively disseminate information, address misconceptions, and promote positive behavior change among young individuals in their efforts to prevent HIV transmission.

### 4.20. Policy and advocacy efforts

Policy and advocacy efforts play a crucial role in shaping the landscape of HIV prevention among youths, influencing legislation, resource allocation, and creating an enabling environment for effective interventions.^[141]^ Advocating for the development and implementation of policies that support comprehensive sexual education, access to healthcare, and HIV prevention services for youths. Lobbying for increased funding and resource allocation towards HIV prevention programs targeted at youths, ensuring sustainable support for interventions.^[142]^ Advocating for legal reforms that protect the rights of young individuals, address discrimination, and promote inclusive and supportive environments for HIV prevention.^[143]^ Campaigning for the integration of comprehensive sexual education into school curricula, ensuring that accurate and age-appropriate information is provided to youths. Influencing policies that improve access to youth-friendly healthcare services, including SRH services, HIV testing, and treatment.^[144]^ Advocating for initiatives aimed at reducing stigma and discrimination associated with HIV/AIDS, sexual orientation, and gender identity among youths.^[145]^

Creates an enabling environment by establishing supportive policies, legal frameworks, and resource allocations conducive to effective HIV prevention among youths. Influences policies that enhance access to healthcare, education, and support services, reducing barriers that prevent youths from seeking essential health information and treatment. Advocacy efforts contribute to securing sustainable funding and support for HIV prevention programs, ensuring their continuity and effectiveness.^[146]^ Protects the rights of young individuals, empowers marginalized groups, and promotes their agency in making informed decisions about their sexual health. Influences societal norms by advocating for inclusive policies and campaigns that challenge stigma, discrimination, and harmful beliefs surrounding HIV/AIDS.^[147]^ Affects broader systemic change by influencing policies that have a lasting impact on HIV prevention efforts and the well-being of youths. Policy and advocacy efforts are essential for fostering a conducive environment that supports effective HIV prevention interventions among youths. By advocating for policies that prioritize education, access to services, rights protection, and stigma reduction, these efforts contribute significantly to creating sustainable change and positively impacting the health and well-being of young individuals in relation to HIV prevention.^[148]^ Implementing and sustaining these comprehensive preventive strategies require collaboration among governments, NGOs, healthcare providers, educators, communities, and young people themselves. Tailoring interventions to specific cultural contexts and continuously evaluating and adapting strategies based on evidence-based approaches are critical in effectively combating HIV among youths.

Preventive Measures against HIV Among Uganda’s Youth

### 4.21. Strategies

Uganda has implemented CSE programs in schools and communities to provide young people with accurate information on HIV transmission, prevention methods, and reproductive health. These programs aim to empower youth to make informed decisions about their SRH, including delaying sexual debut and practicing safer sex. The government and non-governmental organizations (NGOs) distribute free condoms and promote their use among sexually active youth as a key prevention strategy. Condom promotion campaigns target schools, universities, workplaces, and entertainment venues to increase awareness and accessibility. Uganda has scaled up HIV testing and counseling services to reach youth in various settings, including health facilities, schools, and community outreaches. HIV Testing and Counseling (HTC) services offer voluntary testing, counseling, and linkage to care and treatment for those diagnosed with HIV. To address barriers to accessing HIV prevention and care services, Uganda has established youth-friendly health facilities equipped with trained staff, confidential services, and tailored interventions to meet the unique needs of young people. Behavior Change Communication (BCC) campaigns utilize mass media, social media, peer educators, and community outreach to disseminate messages promoting HIV prevention behaviors, including abstinence, faithfulness, and condom use.^[37–41]^

### 4.22. Implementation

The implementation of HIV prevention strategies among Uganda’s youth involves collaboration among government agencies, NGOs, civil society organizations, healthcare providers, educators, and community leaders. Key stakeholders work together to develop, implement, and monitor prevention programs, ensuring they are culturally appropriate, evidence-based, and responsive to the needs of young people. Implementation efforts prioritize reaching marginalized and vulnerable populations, including adolescent girls, young men who have sex with men, and out-of-school youth.^[41–43]^

### 4.23. Effectiveness

While progress has been made in HIV prevention efforts among Uganda’s youth, challenges persist in achieving optimal effectiveness. Despite increased access to prevention services, HIV knowledge gaps, stigma, gender inequalities, and socio-economic factors continue to hinder prevention efforts. Moreover, inconsistent funding, weak health systems, and competing health priorities pose challenges to sustaining and scaling up prevention programs. Continuous monitoring and evaluation are essential to assess the impact of interventions, identify gaps, and refine strategies to improve effectiveness.^[44,45]^

### 4.24. Challenges and barriers

Addressing HIV prevention among youths faces several challenges and barriers that hinder effective interventions.^[149]^ Deep-rooted societal stigma surrounding HIV/AIDS can lead to discrimination against those affected, hindering open discussion and access to healthcare.^[150]^ Inadequate or absent comprehensive sexual education in schools or communities contributes to misinformation and a lack of awareness among youths. Economic disparities limit access to healthcare, education, and resources, making marginalized youths more vulnerable to HIV transmission. Insufficient availability or accessibility of youth-friendly healthcare services and HIV testing centers hinder young individuals from seeking necessary care.^[108]^ Deeply ingrained gender norms that limit girls’ autonomy and control over their sexual health decisions. Insufficient funding and resources allocated to youth-focused HIV prevention programs limit their reach and effectiveness.^[108]^ Peer pressure, early sexual debut, and engaging in risky behaviors increase the likelihood of HIV transmission among youths.^[151]^ Disparities in access to technology and digital resources may limit the effectiveness of innovative technology-based HIV prevention strategies among all youths. Youths affected by conflict, displacement, or migration often face additional challenges in accessing healthcare and preventive services. Absence of policies or legal frameworks supporting comprehensive sexual education and youth-friendly healthcare services. Resistance to adopting safer sexual practices due to cultural beliefs, misinformation, or peer influence. Challenges in delivering culturally sensitive HIV prevention messages that resonate with diverse communities and languages.^[152]^ Addressing these challenges requires a multifaceted approach involving collaborative efforts among governments, policymakers, healthcare providers, educators, community leaders, and youths themselves. Tailoring interventions to specific cultural contexts, advocating for supportive policies, and continuously evaluating and adapting strategies are crucial in overcoming these barriers and enhancing HIV prevention efforts among youths.

### 4.25. Successes in HIV prevention among youths

Greater awareness about HIV/AIDS among youths due to improved access to information, comprehensive sexual education programs, and outreach efforts.^[153]^ Significant advancements in HIV treatment options and care have improved health outcomes and reduced transmission rates among those diagnosed. Utilization of technology and social media platforms for disseminating HIV prevention messages, reaching a wider audience of youths, and encouraging engagement. Empowerment of young individuals through youth-led initiatives, peer education programs, and the involvement of young advocates in shaping HIV prevention strategies.^[154]^

Enhanced access to HIV testing services, including innovative approaches such as self-testing kits, promoting early diagnosis and linkage to care. Increased community engagement, mobilization, and support networks that create supportive environments for youths affected by HIV/AIDS.

### 4.26. Lessons learned

Holistic and comprehensive approaches that address socio-economic, cultural, and structural factors are essential for effective HIV prevention among youths.^[155]^ Importance of tailoring interventions to specific cultural contexts, taking into account diverse beliefs, practices, and social norms among different youth populations. Engaging youths in program design and implementation empowers them to take ownership, ensures relevance, and encourages sustainability of interventions. Sustained efforts and continuity of interventions are crucial for long-term impact, requiring consistent funding, support, and evaluation. Strategies to combat stigma and discrimination are pivotal for creating supportive environments that encourage youths to seek testing and treatment. Utilizing innovative approaches, such as technology-driven campaigns, peer-led education, and creative communication methods, can effectively engage youths. Policy reforms and advocacy efforts are instrumental in creating an enabling environment and allocating resources for successful HIV prevention programs.^[156]^ Learning from both successes and challenges, implementing evidence-based strategies, fostering collaborations among stakeholders, and continuously adapting interventions based on lessons learned are key for sustaining and enhancing successful HIV prevention efforts among youths.

## 5. Recommendations to various stakeholders for preventing HIV Among Uganda’s youth

### 5.1. Government

Increase funding allocation to HIV prevention programs targeting youth, ensuring sustained investment in comprehensive prevention efforts. Strengthen policy frameworks and legislation to support evidence-based HIV prevention interventions, including comprehensive sexuality education and youth-friendly health services. Enhance coordination and collaboration among government ministries, departments, and agencies to ensure a cohesive and integrated approach to HIV prevention. Address socio-economic factors driving HIV vulnerability among youth, such as poverty, gender inequality, and lack of access to education and employment opportunities.

### 5.2. Clinicians

Provide culturally sensitive and youth-friendly HIV testing, counseling, and treatment services, ensuring confidentiality and respect for patients’ rights. Offer comprehensive SRH services, including contraception and pre-exposure prophylaxis (PrEP) for HIV prevention, as part of routine care for youth. Advocate for the integration of HIV prevention into primary healthcare services and community-based initiatives to reach youth in diverse settings. Stay updated on current HIV prevention guidelines and best practices, and actively engage in capacity-building initiatives to enhance HIV prevention knowledge and skills.

### 5.3. Patients

Take proactive steps to protect themselves from HIV by practicing safer sex, using condoms consistently and correctly, and limiting the number of sexual partners. Seek HIV testing and counseling services regularly, especially if sexually active or engaging in high-risk behaviors, and adhere to recommended prevention measures. Engage in open and honest communication with healthcare providers about sexual health concerns, including HIV risk factors and prevention options. Access and utilize youth-friendly health services for comprehensive SRH care, including HIV prevention, testing, and treatment.

### 5.4. Educational institutions

Integrate CSE into school curricula at all levels, providing age-appropriate information on HIV prevention, sexual health, and reproductive rights. Train teachers and school counselors to deliver CSE programs effectively, fostering an inclusive and supportive learning environment that promotes healthy behaviors and positive attitudes toward sexuality. Establish peer education programs and youth-led initiatives to empower students to become advocates for HIV prevention and sexual health promotion within their schools and communities. Provide access to youth-friendly health services on school campuses or through partnerships with local healthcare facilities, ensuring students can access confidential and non-judgmental care.

### 5.5. Public health

Conduct research and surveillance to monitor HIV trends and identify emerging issues among Uganda’s youth, informing evidence-based decision-making and program planning. Implement targeted interventions to reach key populations at higher risk of HIV infection, including adolescent girls, young men who have sex with men, and out-of-school youth. Scale up community-based HIV prevention initiatives, leveraging existing social networks, community organizations, and faith-based groups to deliver tailored interventions. Promote community engagement and participatory approaches to HIV prevention, empowering local stakeholders to take ownership of prevention efforts and drive sustainable change.

### 5.6. Global health

Support Uganda’s efforts to strengthen HIV prevention programs through technical assistance, capacity-building initiatives, and financial support. Advocate for increased funding and resources for HIV prevention among youth, highlighting the importance of investing in evidence-based interventions to achieve sustainable impact. Foster collaboration and knowledge-sharing among global health stakeholders, facilitating the exchange of best practices, innovations, and lessons learned in HIV prevention programming. Prioritize youth voices and perspectives in global health policy and decision-making processes, ensuring their needs and priorities are adequately represented and addressed.

### 5.7. Parents

Engage in open and non-judgmental communication with their children about sexual health, HIV prevention, and responsible decision-making. Provide accurate and age-appropriate information about HIV transmission, prevention methods, and the importance of practicing safer sex. Encourage their children to delay sexual debut and to seek guidance from trusted adults if they have questions or concerns about sexual health. Advocate for CSE in schools and communities, emphasizing the importance of holistic sexual health education.

### 5.8. Youths

Take responsibility for their sexual health by practicing safer sex, using condoms consistently and correctly, and reducing the number of sexual partners. Seek information and support from trusted sources, such as healthcare providers, school counselors, and peer educators, regarding HIV prevention and sexual health. Get tested for HIV regularly and encourage friends and peers to do the same, promoting a culture of testing and knowing one’s status. Advocate for access to youth-friendly health services and comprehensive SRH education in schools and communities.

### 5.9. Community leaders

Provide leadership and support for HIV prevention initiatives in their communities, fostering a culture of acceptance, inclusion, and support for individuals affected by HIV/AIDS. Mobilize community resources and partnerships to address social determinants of HIV vulnerability among youth, including poverty, gender inequality, and stigma. Establish community-based HIV prevention programs and outreach efforts targeting young people, leveraging existing networks and platforms for maximum reach and impact. Promote positive social norms and behaviors related to sexual health and HIV prevention, challenging harmful stereotypes and attitudes that contribute to HIV stigma and discrimination.

## 6. Conclusion

In conclusion, HIV prevention among youths requires a multifaceted and comprehensive approach that addresses various socio-economic, cultural, and structural factors influencing vulnerability to HIV transmission. Overcoming challenges and building on successes in HIV prevention efforts among young individuals involves a combination of strategies, lessons learned, and continuous adaptations.

While successes in HIV prevention efforts among youths are evident, challenges such as stigma, inadequate education, limited resources, and cultural barriers persist. Addressing these challenges requires continued collaboration, innovative approaches, and sustained commitment from governments, organizations, communities, and young individuals themselves. Learning from successes and lessons learned, it’s pivotal to tailor interventions, engage youths in decision-making processes, and prioritize initiatives that create an enabling environment supportive of HIV prevention. By investing in these efforts and continuously evolving strategies, it’s possible to mitigate the impact of HIV/AIDS among youths and create a healthier, more informed, and empowered generation.

## 7. Recommendations

Advocate for and implement comprehensive sexual education programs in schools and communities, ensuring they cover diverse aspects of sexual health, including HIV prevention, consent, healthy relationships, and gender equality. Improve access to youth-friendly healthcare services by expanding facilities, training healthcare providers in youth-specific needs, and ensuring confidentiality and non-judgmental care. Encourage community involvement and peer support initiatives that create safe spaces for youths to access information, share experiences, and receive guidance on sexual health matters. Continue leveraging technology and social media platforms for innovative HIV prevention campaigns, using interactive and engaging content tailored to the preferences of young individuals. Advocate for policies that support youth-focused HIV prevention, including increased funding for programs, integration of comprehensive sexual education into curricula, and legal reforms that protect youths’ rights. Implement programs that address socioeconomic determinants such as poverty, gender inequality, and access to education, as these influence vulnerability to HIV transmission among youths. Launch campaigns to reduce stigma, discrimination, and misconceptions surrounding HIV/AIDS, promoting tolerance, acceptance, and inclusion of individuals affected by the virus. Regularly assess the effectiveness of interventions through monitoring, evaluation, and research to identify areas of improvement and ensure the impact of HIV prevention efforts. Offer capacity-building programs and training sessions for educators, healthcare providers, and community workers involved in youth-focused HIV prevention, enhancing their skills and knowledge. Actively involve young individuals in the design, implementation, and evaluation of HIV prevention programs, ensuring their voices and perspectives are heard and valued. By implementing these recommendations, alongside sustained commitment, collaboration among stakeholders, and continued innovation, it’s possible to strengthen HIV prevention efforts among youths and work towards creating a generation empowered with the knowledge and resources to prevent HIV transmission and lead healthy lives.

## Author contributions

**Conceptualization:** Emmanuel Ifeanyi Obeagu.

**Methodology:** Emmanuel Ifeanyi Obeagu, Getrude Uzoma Obeagu.

**Supervision:** Emmanuel Ifeanyi Obeagu.

**Validation:** Emmanuel Ifeanyi Obeagu, Getrude Uzoma Obeagu.

**Visualization:** Emmanuel Ifeanyi Obeagu, Getrude Uzoma Obeagu.

**Writing – original draft:** Emmanuel Ifeanyi Obeagu, Getrude Uzoma Obeagu.

**Writing – review & editing:** Emmanuel Ifeanyi Obeagu, Getrude Uzoma Obeagu.
